# Null Wave Front and Ryu–Takayanagi Surface

**DOI:** 10.3390/e22111297

**Published:** 2020-11-14

**Authors:** Jun Tsujimura, Yasusada Nambu

**Affiliations:** Department of Physics, Graduate School of Science, Nagoya University, Chikusa, Nagoya 464-8602, Japan; nambu@gravity.phys.nagoya-u.ac.jp

**Keywords:** Ryu–Takayanagi surface, null geodesics, wave front, extremal surface, wave optics

## Abstract

The Ryu–Takayanagi formula provides the entanglement entropy of quantum field theory as an area of the minimal surface (Ryu–Takayanagi surface) in a corresponding gravity theory. There are some attempts to understand the formula as a flow rather than as a surface. In this paper, we consider null rays emitted from the AdS boundary and construct a flow representing the causal holographic information. We present a sufficient and necessary condition that the causal information surface coincides with Ryu–Takayanagi surface. In particular, we show that, in spherical symmetric static spacetimes with a negative cosmological constant, wave fronts of null geodesics from a point on the AdS boundary become extremal surfaces and therefore they can be regarded as the Ryu–Takayanagi surfaces. In addition, from the viewpoint of flow, we propose a wave optical formula to calculate the causal holographic information.

## 1. Introduction

It is well known that the entanglement entropy (EE) of conformal field theory (CFT) can calculate in a corresponding gravity theory by the Ryu–Takayanagi (RT) formula [1,2] in AdS/CFT correspondence [3,4]. In general, although the EE of quantum field theory is not easy to calculate, the RT formula tells that the EE $S_{A}$ of a region *A* in CFT can calculated as the area of the minimal bulk surface $M_{A}$ homologous to *A* (RT surface):

(1) $$\begin{array}{c} S_{A}=\frac{\mathrm{Area}\left(M_{A}\right)}{4G_{N}}, \end{array}$$

where $G_{N}$ is the Newton constant of gravitation. This relation promotes informational theoretical analysis of AdS/CFT correspondence. By regarding the geometry of a bulk as a tensor network, it implements quantum error correcting code of boundary CFT [5] or MERA [6], subregion correspondence, which is proposed for a reduced density matrix [7].

From this point of view, it is better to regard the RT formula as a flow proposed by the paper [8]. The authors introduced “bit threads” which are an equivalent concept to the RT surface geometrically. The bit threads are defined as a bounded divergenceless vector field $v^{\mu }$

(2) $$\begin{array}{c} \nabla_{\mu }v^{\mu }=0,\;\left|v\right|\leq C, \end{array}$$

and it maximizes its flux on a boundary area *A*. The property that the maximal total flux of $v^{\mu }$ through the area *A* is equal to the area of the RT surface is proved by the max-flow min-cut theorem [8]. The bit threads give an intuitive picture that a vector field carrying information of the boundary propagates in the bulk, and the bulk region stores information of the boundary. Considering the bulk reconstruction, the bit threads have better properties than the RT surface although it is geometrically equivalent concept to the RT surface. Recall that the RT surface cannot reach the neighborhood of the black hole horizon. On the other hand, the bit threads can probe such a bulk regions. See other benefits of considering the bit threads for example [9].

As a similar concept of holographic EE, a causal holographic information $\chi_{A}$ [10] was defined by

(3) $$\begin{array}{c} \chi_{A}=\frac{\mathrm{Area}\left(\Xi_{A}\right)}{4G_{N}}, \end{array}$$

where $\Xi_{A}$ is the causal information surface of *A* defined as follows. Let us consider the bulk domain of influence of the boundary domain of dependence of a region *A*. Then, the causal information surface $\Xi_{A}$ is the intersection of the future and past directed null surfaces characterizing such a bulk domain of influence. In a static system, we can regard the causal information surface $\Xi_{A}$ as a null wave front anchored on ∂A. A causal holographic information $\chi_{A}$ is known as an upper bound of EE of *A*. That is, $S_{A}\leq \chi_{A}$ as discussed in [10].

When does the equality $S_{A}=\chi_{A}$ hold? In this paper, we consider a sufficient and necessary condition to hold it from a viewpoint of flow. By its definition, the causal holographic information $\chi_{A}$ has a natural flow as a null geodesic congruence. Since RT surface is extremal surface [11,12], we are to clarify the condition for a null wavefront to be extremized. We will see that a null wavefront becomes an extremal surface if and only if the shear of the corresponding null congruence vanishes. For example, in spherical symmetric static spacetimes, owing to its axisymmetry of the configuration, wave fronts of null rays emitted from a point on the AdS boundary are extremal surfaces as long as they propagate in the vacuum region.

If $S_{A}=\chi_{A}$, we can regard the wave front as the RT surface and the corresponding null rays as bit threads. In this picture, we can calculate the EE of CFT by counting the number of such null rays. This method is also valid for wave optical calculation using the flux of a massless scalar field. The flux based calculation method suggests a picture that information prepared on the boundary side spreads to the bulk as null rays.

The structure of this paper is as follows. In Section 2, we demonstrate the correspondence between the RT surface and the wave front of the null rays in the BTZ spacetime. In Section 3, we state the detail of our proposal and show it in spherically symmetric static spacetimes with a negative cosmological constant. In Section 4, we introduce the flux formula to calculate the causal holographic information by counting the number of null rays. Finally, Section 5 is devoted to the conclusions.

## 2. Null Wave Front and RT Surface

In this section, before going to discuss the general situation, we demonstrate that wave fronts of null rays are the RT surface in the BTZ spacetime.

### 2.1. Ryu–Takayanagi Surface

We derive the equation of the RT surface in the BTZ spacetime [13]

(4) $$\begin{array}{c} ds^{2}=-\left(\frac{r^{2}}{\ell_{\mathrm{AdS}}^{2}}-M\right)dt^{2}+{\left(\frac{r^{2}}{\ell_{\mathrm{AdS}}^{2}}-M\right)}^{-1}dr^{2}+r^{2}d\theta^{2},\;-\pi \leq \theta \leq \pi , \end{array}$$

where *M* is the mass of the black hole and $\ell_{\mathrm{AdS}}$ is the AdS radius. We prepare a region (arc) $-\theta_{\ell }\;\leq \;\theta \;\leq \;\theta_{\ell }$ on the AdS boundary and consider a line anchored to the boundary of this region. The RT surface extremizes the following line area (length) on a constant time slice:

(5) $$\begin{array}{c} \mathrm{Area}\left[r,\frac{dr}{d\theta }\right]=\int_{-\theta_{\ell }}^{\theta_{\ell }}d\theta \sqrt{{\left(\frac{r^{2}}{\ell_{\mathrm{AdS}}^{2}}-M\right)}^{-1}{\left(\frac{dr}{d\theta }\right)}^{2}+r^{2}}. \end{array}$$

**Figure 1:**
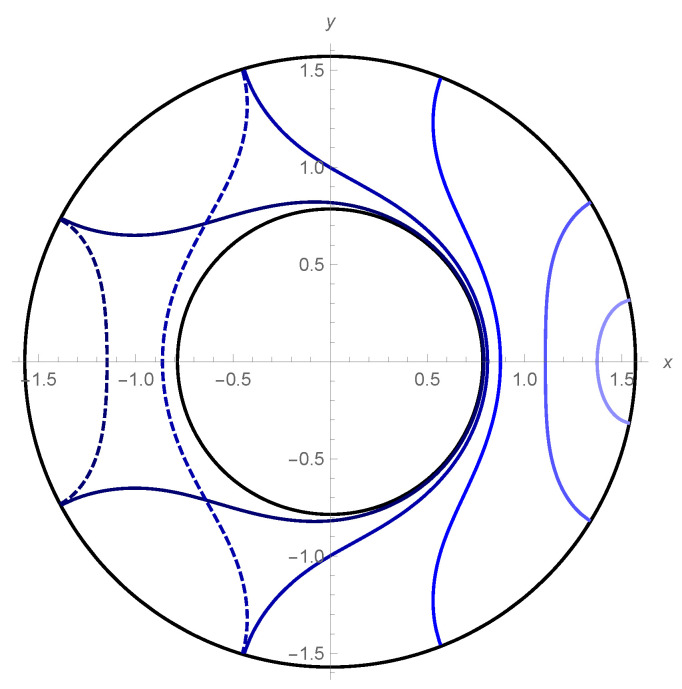
The RT surface in the BTZ spacetime ($M=\ell_{\mathrm{AdS}}=1$) with coordinates (x,y)=(ρcosθ,ρsinθ), $\rho :=\ell_{\mathrm{AdS}}\arctan\left(r/\ell_{\mathrm{AdS}}\right)$. Each blue line is parametrized by $r_{\min}=1.01,1.05,1.2,2.0,5.0$. For large interval $2\theta_{\ell }$ on the AdS boundary, dotted lines become minimal surfaces.

The equation of the RT surface $r=r_{\mathrm{RT}}\left(\theta \right)$ is the solution of the Euler–Lagrange equation obtained by variation of Area[r,dr/dθ] with respect to *r*, and it is

(6) $$\begin{array}{c} r_{\mathrm{RT}}\left(\theta \right)=\frac{\sqrt{M}\;r_{\min}\;\mathrm{sech}\left(\sqrt{M}\;\theta \right)}{\sqrt{M-r_{\min}^{2}/\ell_{\mathrm{AdS}}^{2}\tanh^{2}\left(\sqrt{M}\;\theta \right)}}, \end{array}$$

where $r_{\min}:=r_{\mathrm{RT}}\left(\theta =0\right)$ denotes the minimum of *r* (see Figure 1). Note that $\theta_{\ell }=\theta \left(r=\infty \right)=\left(1/\sqrt{M}\right)\mathrm{arctanh}\left(\sqrt{M}\ell_{\mathrm{AdS}}/r_{\min}\right)$.

The entanglement entropy of CFT on the AdS boundary for an arc $\left|\theta \right|\leq \theta_{\ell }$ is obtained by substituting (6) into (5):

(7) $$\begin{array}{c} \mathrm{Area}\left(M_{\ell }\right)=2\ell_{\mathrm{AdS}}\log\left(\frac{2\ell_{\mathrm{AdS}}}{\epsilon \sqrt{M}}\sinh\left(\sqrt{M}\;\theta_{\ell }\right)\right)+O\left(\epsilon \right), \end{array}$$

where the cutoff is introduced by $\epsilon :=\ell_{\mathrm{AdS}}^{2}/r\;\left(r\to \infty \right)$. Now let us consider CFT with inverse temperature β on $S^{1}$. The circumference of the circle is assumed to be *C* and we prepare an arc $\left|\theta \right|\leq \theta_{\ell }$ with the arc length $\ell =C\theta_{\ell }/\pi$ on it. Then, it is possible to write down (7) using only CFT quantities. By dividing Equation (7) with $4G_{N}$, using the Brown–Henneaux formula $c=3\ell_{\mathrm{AdS}}/\left(2G_{N}\right)$ [14] and AdS/CFT dictionary $\beta /C=1/\sqrt{M}$, we obtain the correct EE formula of thermal state of CFT on $S^{1}$ [15,16] after rescaling the cutoff ϵ:

(8) $$S_{A}=\frac{c}{3}\log\left(\frac{\beta }{\pi \epsilon }\sinh\left(\frac{\pi \ell }{\beta }\right)\right).$$

### 2.2. Null Rays and Wave Front

We consider null rays emitted from a point on the AdS boundary and their wave fronts. Our purpose is to find out the relation between wave fronts of null rays and the RT surface. We consider null rays in the spherically symmetric static spacetime

(9) $$ds^{2}=-f\left(r\right)dt^{2}+\frac{dr^{2}}{f(r)}+r^{2}d\Omega_{d-1}^{2},$$

where *d* denotes spatial dimension and $d\Omega_{d-1}^{2}$ is the line element of the unit sphere $S^{d-1}$. We introduce coordinates on $S^{d-1}$ as

(10) $$\left[\begin{array}{c} x_{1}\\ x_{2}\\ x_{3}\\ \vdots \\ x_{d-2}\\ x_{d-1} \end{array}\right]=\left[\begin{array}{c} \cos\psi_{1}\\ \sin\psi_{1}\cos\psi_{2}\\ \sin\psi_{1}\sin\psi_{2}\\ \vdots \\ \sin\psi_{1}\sin\psi_{2}\cdots \sin\psi_{d-2}\cos\psi_{d-1}\\ \sin\psi_{1}\sin\psi_{2}\cdots \sin\psi_{d-2}\sin\psi_{d-1} \end{array}\right]$$

with $0\leq \psi_{1},\cdots \psi_{d-2}\leq \pi ,\;0\leq \psi_{d-1}\leq 2\pi$. The line element on $S^{d-1}$ is

(11) $$d\Omega_{d-1}^{2}=d\psi_{1}^{2}+\sin^{2}\psi_{1}\left(d\psi_{2}^{2}+\sin^{2}\psi_{2}\left(d\psi_{3}^{2}+\cdots \cdots \right)\right).$$

As is well known, in static spherically symmetric spacetimes, trajectories of null geodesics stay on a spatial two-dimensional plane. Thus, we can fix coordinate values of $\psi_{2},\cdots \psi_{d-1}$ and assume the following (2+1)-dimensional metric to investigate wave fronts of null rays emitted from a point:

(12) $$ds^{2}=-f\left(r\right)dt^{2}+\frac{dr^{2}}{f(r)}+r^{2}d\theta^{2},\;\theta :=\psi_{1}.$$

**Figure 2:**
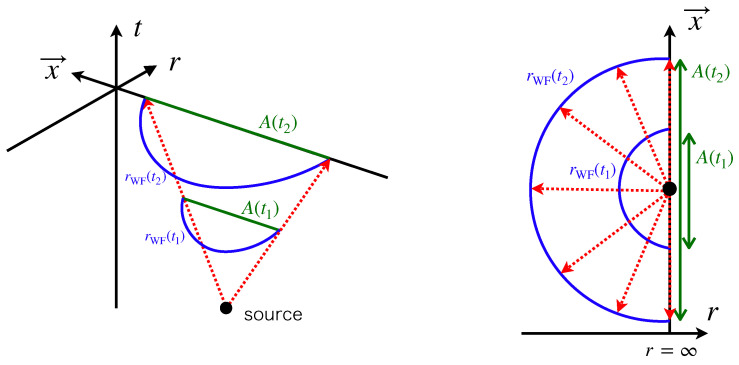
**Left panel**: Axisymmetric configurations of congruence of null rays emitted from a point source on the AdS boundary. The **right panel**: the wavefronts projected onto a t=const. time slice. The coordinate $\vec{x}$ denotes d−1 dimensional space as Equation (10). The blue, red, and green lines represent the wavefronts $r_{\mathrm{WF}}\left(t\right)$, the null rays and subregion A(t), respectively.

In a static spacetime, a wave front of null rays emitted from a point source is defined as a t= constant section of null congruences as depicted in Figure 2, which forms a (d−1)-dimensional surface. Due to the axial symmetry of the configuration, a wave front of null rays is represented as a curve in (r,θ) space in the present situation. The tangent vector of a null ray is

(13) $$k^{\mu }=\left(k^{t},k^{r},k^{\theta }\right)=\left(\frac{dt}{d\lambda },\frac{dr}{d\lambda },\frac{d\theta }{d\lambda }\right),\;-f{\left(\frac{dt}{d\lambda }\right)}^{2}+\frac{1}{f}{\left(\frac{dr}{d\lambda }\right)}^{2}+r^{2}{\left(\frac{d\theta }{d\lambda }\right)}^{2}=0,$$

where λ is the affine parameter. This spacetime has two Killing vectors related to translation of *t* and θ directions and there exist two conserved charges $\omega :=f\left(r\right)\frac{dt}{d\lambda },\;p_{\theta }:=r^{2}\frac{d\theta }{d\lambda }$. Combining with Equation (13), we obtain a trajectory of a null ray as

(14) $$\begin{array}{cc} \theta (r) & =\theta_{0}\pm \int_{r_{0}}^{r}\frac{b}{r^{\prime 2}}{\left(1-f\left(r^{\prime }\right)\frac{b^{2}}{r^{\prime 2}}\right)}^{-\frac{1}{2}}dr^{\prime }, \end{array}$$

(15) $$\begin{array}{cc} t(r) & =t_{0}\pm \int_{r_{0}}^{r}\frac{1}{f(r^{\prime })}{\left(1-f\left(r^{\prime }\right)\frac{b^{2}}{r^{\prime 2}}\right)}^{-\frac{1}{2}}dr^{\prime }, \end{array}$$

(16) $$\begin{array}{cc} \lambda (r) & =\lambda_{0}\pm \frac{1}{\omega }\int_{r_{0}}^{r}{\left(1-f\left(r^{\prime }\right)\frac{b^{2}}{r^{\prime 2}}\right)}^{-\frac{1}{2}}dr^{\prime }, \end{array}$$

where $\left(t_{0},\theta_{0},\lambda_{0}\right)=\left(t\left(r_{0}\right),\theta \left(r_{0}\right),\lambda \left(r_{0}\right)\right)$ and the impact parameter $b:=p_{\theta }/\omega$ is introduced. The sign ± in front of the integral corresponds to the sign of dr/dλ.

For the (2+1)-dimensional BTZ spacetime (4), we can demonstrate explicitly that wave fronts of null rays are the RT surfaces. We obtain equations of null geodesic from (14) and (15) with $\left(t_{0},r_{0},\theta_{0}\right)\;=\;\left(0,\infty ,0\right)$:

(17) $$\begin{array}{cc} \theta (r) & =\frac{1}{\sqrt{M}}\log\left[\frac{\sqrt{r^{2}-b^{2}\left(r^{2}/\ell_{\mathrm{AdS}}^{2}-M\right)}+b\sqrt{M}}{r\sqrt{1-b^{2}/\ell_{\mathrm{AdS}}^{2}}}\right], \end{array}$$

(18) $$\begin{array}{cc} t(\theta ) & =\frac{\ell_{\mathrm{AdS}}}{\sqrt{M}}\mathrm{arctanh}\left(\frac{\ell_{\mathrm{AdS}}}{b}\tanh\left(\sqrt{M}\;\theta \right)\right). \end{array}$$

It is easy to derive a trajectory of a null ray $r=r_{\mathrm{NG}}\left(\theta ,b\right)$ with an impact parameter *b* from (17). On the other hand, the equation of a wave front $r=r_{\mathrm{WF}}\left(\theta ,t\right)$ at a fixed *t* is derived by eliminating the parameter *b* from (17) and (18). After all,

(19) $$\begin{array}{cc} r_{\mathrm{NG}}\left(\theta ,b\right) & =\frac{\sqrt{M}\;b}{\sqrt{1-b^{2}/\ell_{\mathrm{AdS}}^{2}}}\;\mathrm{csch}\left(\sqrt{M}\;\theta \right), \end{array}$$

(20) $$\begin{array}{cc} r_{\mathrm{WF}}\left(\theta ,t\right) & =\frac{\sqrt{M}\ell_{\mathrm{AdS}}\;\coth\left(\sqrt{M}\;t/\ell_{\mathrm{AdS}}\right)\mathrm{sech}\left(\sqrt{M}\;\theta \right)}{\sqrt{1-\coth^{2}\left(\sqrt{M}\;t/\ell_{\mathrm{AdS}}\right)\tanh^{2}\left(\sqrt{M}\;\theta \right)}}. \end{array}$$

**Figure 3:**
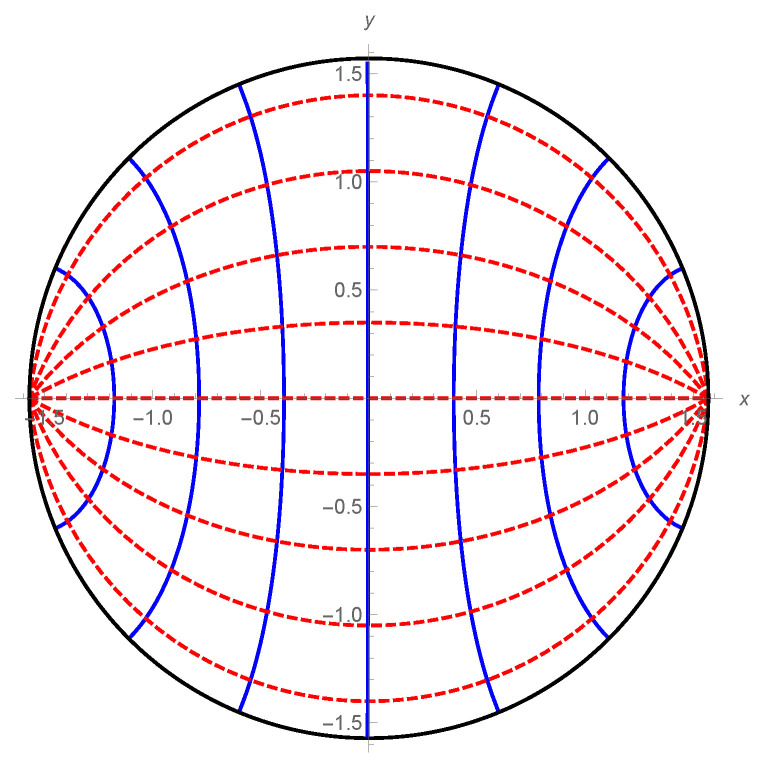
Null rays (dotted red lines) and wave fronts (blue lines) in the pure AdS${}_{2+1}$ spacetime $\left(M=-1,\ell_{\mathrm{AdS}}=1\right)$.

**Figure 4:**
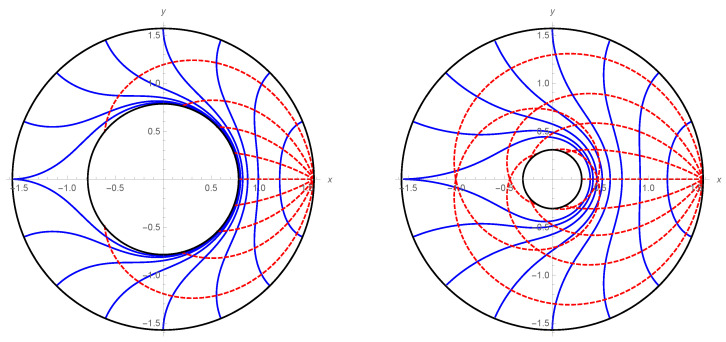
Null rays (dotted red lines) emitted from (r,θ)=(∞,0) and their wave fronts (blue lines) in the BTZ spacetime (**left panel**: $M=\ell_{\mathrm{AdS}}=1$, **right panel**: $M=0.1,\ell_{\mathrm{AdS}}=1$).

For the special case M=−1, the spacetime reduces to the pure AdS. Figure 3 and Figure 4 show null rays and their wave fronts in the pure AdS spacetime and the BTZ black hole spacetime, respectively.

Note that Equation (20) of the wave front is the same as Equation (6) of the RT surface by identifying $r_{\min}=\sqrt{M}\ell_{\mathrm{AdS}}\coth\left(\sqrt{M}t/\ell_{\mathrm{AdS}}\right)$, which represents the elapsed time of a null ray traveling from r=∞ to $r=r_{\min}$. Indeed, this quantity is obtained by taking b=0 in the equation of the null ray (15):

(21) $$\begin{array}{c} t=\int_{\infty }^{r_{\min}}\frac{dr}{r^{2}/\ell_{\mathrm{AdS}}^{2}-M}=\frac{\ell_{\mathrm{AdS}}}{\sqrt{M}}\;\;\mathrm{arctanh}\left(\frac{\sqrt{M}\ell_{\mathrm{AdS}}}{r_{\min}}\right). \end{array}$$

Therefore, we have confirmed that wave fronts of null rays emitted from the AdS boundary coincide with the RT surfaces in the BTZ spacetime. Note that, for sufficient elapse of time after emission of null rays, a self-intersection of the wave front occurs. Then, one might consider that the identification of the wave front as the RT surface becomes ambiguous. However, we do not have to consider such a situation because the “subregion” passed by null rays become bigger than whole boundary region, and then RT formula makes no sense.

## 3. Null Wave Front and Extremal Surface

In this section, based on the observation in the previous section for the BTZ spacetime, we show the following proposition for spherically symmetric static spacetimes with a cosmological constant (no matter fields).

**Proposition** **1.** *Wave fronts of shear free null congruence are extremal surfaces in static spacetime when the affine parameter of null rays goes to infinity.*

**Corollary** **1.** *In static spacetimes with a negative cosmological constant, wave fronts of null rays emitted from the AdS boundary are extremal surfaces if and only if the shear of the null congruence vanishes.*

We adopt the metric (12) with coordinates $x^{\mu }=\left(t,r,\theta ,\cdots \right)$. Let $\xi^{\mu }={\left(\partial_{t}\right)}^{\mu }$ be the time-like Killing vector, $k^{\mu }=dx^{\mu }/d\lambda$ be the tangent vector of null geodesics. We introduce the projection tensor $P^{\mu \nu }=g^{\mu \nu }-\xi^{-2}\xi^{\mu }\xi^{\nu }=\mathrm{diag}\left(0,f,1/r^{2},\cdots \right)$ onto a constant time slice. We denote the tangent vector of null geodesics projected onto the hypersurface as ${\tilde{k}}^{i}=P^{i}{}_{j}\;k^{j}=\left(k^{r},k^{\theta },0,\cdots \right)$. The conserved quantity associated with the Killing vector is $\omega =-\xi^{\mu }k_{\mu }=fk^{t}$ and the norm of the spatial vector ${\tilde{k}}^{i}$ is ${\tilde{k}}^{i}{\tilde{k}}_{i}=\omega^{2}/f$.

We prove the proposition by using the fact that the extremal surface is a surface with zero mean curvature. The mean curvature *H* of a wave front of null rays on a constant time slice is defined by

(22) $$H:=D_{i}{\tilde{n}}^{i},\;{\tilde{n}}^{i}=\frac{{\tilde{k}}^{i}}{\tilde{k}}=\frac{f^{1/2}}{\omega }\left(k^{r},k^{\theta },0,\cdots \right),$$

where ${\tilde{n}}^{i}$ is the unit normal vector of the wave front and $D_{i}=P_{i}{}^{j}\nabla_{j}=\left(\nabla_{r},\nabla_{\theta },\cdots \right)$ is the covariant derivative on a constant time slice. Then,

(23) $$\begin{array}{cc} H & =D_{i}\left(\frac{f^{1/2}}{\omega }k^{i}\right)=\frac{1}{f^{-1/2}\sqrt{h}}\partial_{i}\left(\frac{f^{1/2}}{\omega }f^{-1/2}\sqrt{h}\;k^{i}\right)\\ \; & =\frac{f^{1/2}}{\omega \sqrt{h}}\left(\partial_{r}\left(\sqrt{h}\;k^{r}\right)+\partial_{\theta }\left(\sqrt{h}\;k^{\theta }\right)\right), \end{array}$$

where $\sqrt{h}=r^{d-1}$ comes from determinant of the metric on $S^{d-1}$. On the other hand, the expansion of a null congruence is

(24) $$\begin{array}{cc} \Theta & =\nabla_{\mu }k^{\mu }=\frac{1}{\sqrt{h}}\partial_{\mu }\left(\sqrt{h}\;k^{\mu }\right)\\ \; & =\frac{1}{\sqrt{h}}\left(\partial_{r}\left(\sqrt{h}\;k^{r}\right)+\partial_{\theta }\left(\sqrt{h}\;k^{\theta }\right)\right). \end{array}$$

Therefore, $H=(f^{1/2}/\omega )\Theta$ and the mean curvature *H* of a wave front is proportional to the expansion of the null geodesic congruence. The expansion Θ along a null geodesic obeys the Raychaudhuri equation

(25) $$\frac{d\Theta }{d\lambda }=-\frac{\Theta^{2}}{d-1}-R_{\mu \nu }k^{\mu }k^{\nu }.$$

In the present case, as the congruence of null geodesics has axial symmetry, the shear and the rotation of the congruence do not appear in this equation. For vacuum spacetimes with a cosmological constant, the term with the Ricci curvature disappears. Then, the solution of Equation (25) is $\Theta \left(\lambda \right)\;=\;\left(d\;-\;1\right)/\left(\lambda \;-\;\lambda_{0}\right)$, where $\lambda_{0}$ is the affine parameter at the source. Thus, the expansion goes to zero as the affine parameter goes to infinity, and the mean curvature of the wave front is zero and is the extremal surface. Therefore, the proposition is proved. As an example of this proposition, let us consider a wave front in the Minkowski spacetime. A spherical wave front emitted from a point source placed at the spatial infinity becomes plane wave, which is zero mean curvature surface in the Minkowski spacetime. However, in this case, the coordinate time (15) becomes infinite when a wave front of null rays arrives at an observer.

Asymptotically AdS spacetimes are peculiar because they have the timelike boundary. We consider the pure AdS spacetime of which metric function is given by $f\left(r\right)=1+r^{2}/\ell_{\mathrm{AdS}}^{2}$. As $f\approx r^{2}/\ell_{\mathrm{AdS}}^{2}$ in the vicinity of the AdS boundary, the affine parameter of null rays (16) from the AdS boundary $r_{0}=\ell_{\mathrm{AdS}}^{2}/\epsilon ,\epsilon \to 0$ diverges as

(26) $$\begin{array}{c} \lambda \left(r\right)\approx \frac{1}{\omega }\int_{r}^{\ell_{\mathrm{AdS}}^{2}/\epsilon }{\left(1-\frac{b^{2}}{\ell_{\mathrm{AdS}}^{2}}\right)}^{-1/2}dr=\frac{\ell_{\mathrm{AdS}}^{2}/\epsilon -r}{\omega \sqrt{1-b^{2}/\ell_{\mathrm{AdS}}^{2}}}\to \infty . \end{array}$$

On the other hand, the coordinate time (15) converges as

(27) $$\begin{array}{c} t\left(r\right)\approx \int_{r}^{\ell_{\mathrm{AdS}}^{2}/\epsilon }dr\frac{\ell_{\mathrm{AdS}}^{2}}{r^{2}}{\left(1-\frac{b^{2}}{\ell_{\mathrm{AdS}}^{2}}\right)}^{-1/2}=\frac{\ell_{\mathrm{AdS}}^{2}}{\sqrt{1-b^{2}/\ell_{\mathrm{AdS}}^{2}}}\;\frac{1}{r}. \end{array}$$

This property also holds for general asymptotically AdS spacetimes because they have the same metric in the vicinity of the AdS boundary as the pure AdS spacetime. After all, we conclude that, for static spherical symmetric asymptotically AdS spacetimes, wave fronts of a null geodesic congruence emitted from a point source on the AdS boundary are extremal surfaces.

We can see that the condition for $S_{A}=\chi_{A}$ is that the shear of null congruence vanishes. To satisfy this, we need the strong symmetry for the spacetime and the wavefront.

## 4. Flux Formula

**Figure 5:**
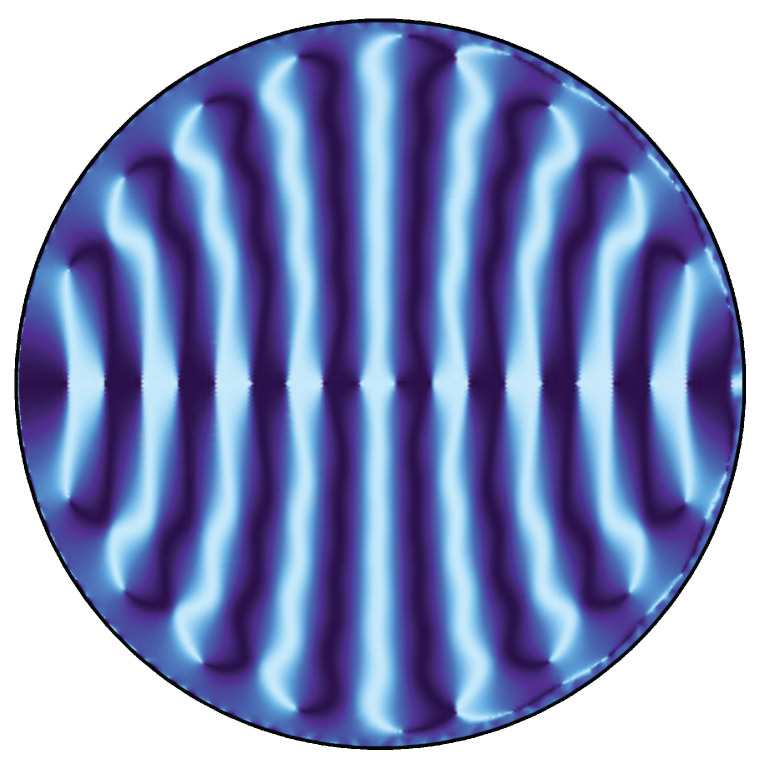
Wave pattern of the monochromatic massless scalar field with ω=20 in the AdS spacetime. The real part of ϕ/|ϕ| is shown.

**Figure 6:**
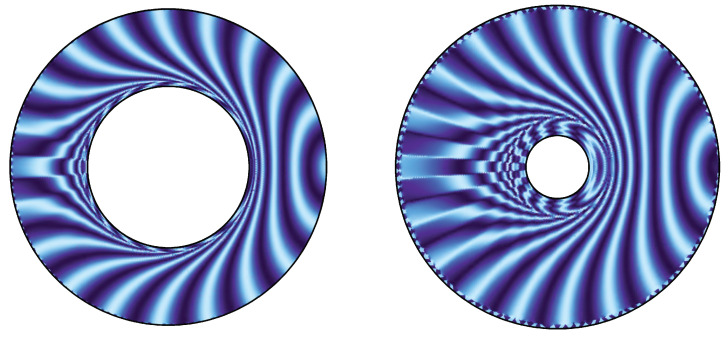
Wave pattern of the massless scalar field with ω=20 (Re ϕ/|ϕ|) in the BTZ spacetime with M=1 (**left panel**) and M=0.1 (**right panel**).

Based on the idea presented in the previous section, we can understand null rays as a natural flow characterizing the EE of the dual CFT if $S_{A}=\chi_{A}$. Hence, a congruence of null rays is one of the bit threads described in Section 1. This makes us conceive a picture that null rays propagate in the bulk with information of the AdS boundary. This picture suggests that the EE can be calculable by counting the number of null rays. In this section, we reformulate the RT formula in terms of the wave optics. Concepts of wave fronts and the flux of null rays are naturally derived as the eikonal limit of wave optics. As an application of wave optics to black hole spacetimes, Refs. [17,18,19] investigate image formation of the photon sphere of black holes. In this paper, we focus on the structure of wave fronts of a massless scalar field. For the monochromatic massless scalar field with time dependence $\propto e^{-i\omega t}$, we present wave patterns in Figure 5 and Figure 6 (see details in the Appendix A. We also show a wave pattern for $S_{A}\neq \chi_{A}$ case in Appendix A). They show wave fronts from a point wave source on the AdS boundary (see Figure 3 and Figure 4 for corresponding wave fronts in the geometrical optics).

For the massless scalar field $\phi (x^{\mu })$ obeying the Klein–Gordon equation $\phi \;=\;{\left(\sqrt{-g}\right)}^{-1}\partial_{\mu }\left(\sqrt{-g}g^{\mu \nu }\partial_{\nu }\phi \right)=0$, the WKB form of the wave function is

(28) $$\phi \left(x^{\mu }\right)=a\left(x^{\mu }\right)\exp\left[iS(x^{\mu })\right],$$

where *a* and *S* are real functions. In the eikonal limit, they obey

(29) $$\begin{array}{cc} \; & g^{\mu \nu }\nabla_{\mu }S\nabla_{\nu }S=0, \end{array}$$

(30) $$\begin{array}{cc} \; & \nabla_{\mu }\left(a^{2}\nabla^{\mu }S\right)=0. \end{array}$$

The Equation (29) is the Hamilton–Jacobi (HJ) equation and Equation (30) represents conservation of the Klein–Gordon current $J^{\mu }=\left(1/2i\right)\left(\phi^{*}\partial^{\mu }\phi -\phi \partial^{\mu }\phi^{*}\right)$. In terms of the wave vector $k_{\mu }=\partial_{\mu }S$, which defines the tangent of null rays,

(31) $$g_{\mu \nu }k^{\mu }k^{\nu }=0,\;\nabla_{\mu }\left(a^{2}k^{\mu }\right)=0.$$

For the stationary case, the phase function *S* can be written as S=−ωt+W(r,θ),

(32) $$\begin{array}{cc} \; & {\tilde{k}}^{i}=\left(k^{r},k^{\theta },0,\cdots \right)=\left(fW_{r},\frac{1}{r^{2}}W_{\theta },0,\cdots \right),\;{\tilde{k}}^{i}{\tilde{k}}_{i}=\frac{\omega^{2}}{f}, \end{array}$$

(33) $$\begin{array}{cc} \; & f^{-1/2}D_{i}\left(a^{2}f^{1/2}{\tilde{k}}^{i}\right)=0. \end{array}$$

**Figure 7:**
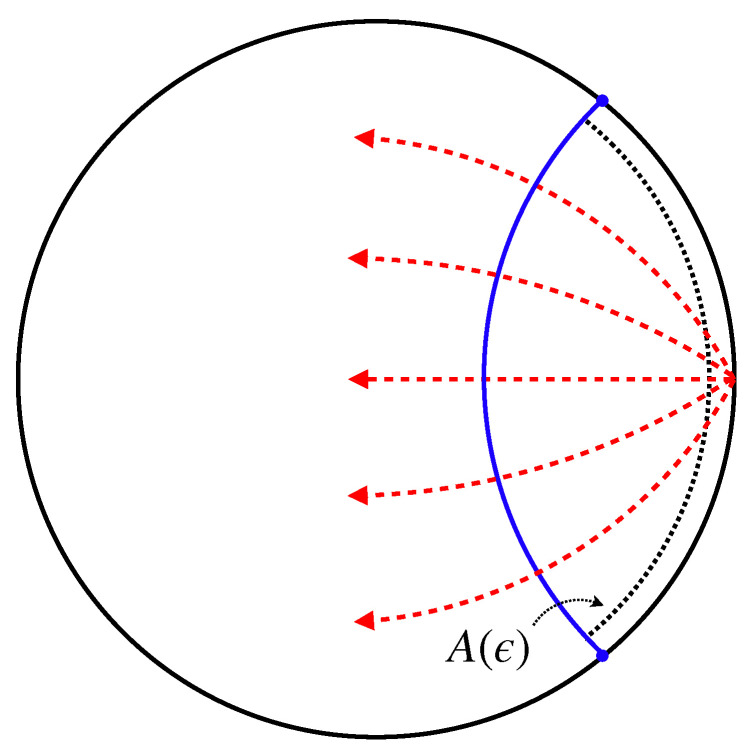
Null rays (red dotted lines) emitted from a point on the AdS boundary pass through the screen A(ϵ) placed at $r=\ell_{\mathrm{AdS}}^{2}/\epsilon$ (dotted line). As the null rays are orthogonal to the wave front (blue line), the number of null rays is proportional to the area of the RT surface.

Here, ${\tilde{k}}^{i}$ represents the tangent vector of null rays projected on a constant time slice. We can write the solution of (30) as

(34) $$a\left(\lambda ,\chi \right)=a\left(\lambda_{0},\chi \right)\exp\left(-\frac{1}{2}\int_{\lambda_{0}}^{\lambda }d\lambda \;\Theta \left(\lambda \right)\right),\;\Theta =\nabla_{\mu }k^{\mu },$$

where the integral is along a null ray (with respect to the affine parameter λ) and χ denotes a coordinate distinguishing different geodesics. As the expansion of null congruence from the AdS boundary is zero, the amplitude a(λ,χ) is conserved along a null ray and independent of λ. Furthermore, for a point source isotropically emitting null rays, *a* is independent of χ and can assume to be constant. Thus, (33) implies

(35) $$D_{i}{\tilde{n}}^{i}=0,\;{\tilde{n}}^{i}=\frac{f^{1/2}}{\omega }{\tilde{k}}^{i},\;{\tilde{n}}^{i}{\tilde{n}}_{i}=1,$$

and ${\tilde{n}}^{i}$ is divergenceless normalized vector field. A vector field $v^{\mu }=\left(0,{\tilde{n}}^{i}\right)$ is one realization of the bit threads satisfying Equation (2). Notice that this construction highly depends on the stationarity of the spacetime. The wave front is the surface with the unit normal ${\tilde{n}}^{i}$, and is the extremal surface. The number of null rays passing through the wave front $E_{A}$, which is the extremal surface homologous to the region *A* on the AdS boundary, is

(36) $$\mathrm{Area}\left(E_{A}\right)=\int_{E_{A}}{\tilde{n}}^{i}d\Sigma_{i},\;d\Sigma_{i}={\tilde{n}}_{i}\sqrt{h}\;d^{d-1}\sigma ,$$

where $\sqrt{h}$ denotes determinant of the induced metric on $E_{A}$. Now let us consider the setup shown as Figure 7. We prepare a screen A(ϵ) which is r= constant surface in the bulk. For the regularization, the screen is placed at $r=\ell_{\mathrm{AdS}}^{2}/\epsilon$ near the AdS boundary.

Because ${\tilde{n}}^{i}$ is a divergence free vector field, Equation (36) equals

(37) $$\mathrm{Area}\left(E_{A}\right)=\int_{A(\epsilon )}{\tilde{n}}^{i}d\Sigma_{i}=\frac{1}{\omega }\int_{A(\epsilon )}J^{i}d\Sigma_{i}.$$

This is a formula for area of the RT surface in terms of flux integration of null rays on the screen A(ϵ). As the Klein–Gordon current $J^{i}/\omega =f^{1/2}{\tilde{k}}^{i}/\omega$ represents the number density of null rays, we can regard the Klein–Gordon current as a representation of the amount of information propagating in the bulk spacetime from the AdS boundary.

As a demonstration, we evaluate the right-hand side of this relation for the BTZ spacetime. By fixing the radial coordinate as $r=\ell_{\mathrm{AdS}}^{2}/\epsilon$ in Equation (19), the impact parameter *b* on the screen is

(38) $$\begin{array}{c} b=\frac{\ell_{\mathrm{AdS}}^{2}\sinh\left(\sqrt{M}\;\theta \right)}{\sqrt{\epsilon^{2}M+\ell_{\mathrm{AdS}}^{2}\sinh^{2}\left(\sqrt{M}\;\theta \right)}}. \end{array}$$

From Equation (13), the radial component of the tangent vector of the null ray is

(39) $$\begin{array}{c} \frac{{\tilde{k}}^{r}}{\omega }={\left(1-f\;\frac{b^{2}}{r^{2}}\right)}^{1/2}=\sqrt{1-\left(\frac{r^{2}}{\ell_{\mathrm{AdS}}^{2}}-M\right)\frac{b^{2}}{r^{2}}}, \end{array}$$

and, on the screen,

(40) $${\left.\frac{{\tilde{k}}^{r}}{\omega }\right|}_{A(\epsilon )}=\frac{\sqrt{M}\cosh\left(\sqrt{M}\;\theta \right)}{\sqrt{M+\left(\ell_{\mathrm{AdS}}^{2}/\epsilon^{2}\right)\sinh^{2}\left(\sqrt{M}\;\theta \right)}}.$$

The area element on the screen is

(41) $$\begin{array}{c} {\left.d\Sigma_{r}\right|}_{A(\epsilon )}={\left.r\;n_{r}\;d\theta \right|}_{A(\epsilon )}=rf^{-1/2}d\theta , \end{array}$$

where $n_{r}$ is the radial component of the unit normal to the screen. Thus,

(42) $$f^{1/2}\frac{{\tilde{k}}^{r}}{\omega }{\left.d\Sigma_{r}\right|}_{A(\epsilon )}=r{\left(1-f\;\frac{b^{2}}{r^{2}}\right)}^{1/2}\;\;\;\;d\theta =\frac{\sqrt{M}\ell_{\mathrm{AdS}}\cosh\left(\sqrt{M}\theta \right)d\theta }{\sqrt{\sinh^{2}\left(\sqrt{M}\theta \right)+M\epsilon^{2}/\ell_{\mathrm{AdS}}^{2}}}.$$

Therefore, (37) becomes

(43) $$\begin{array}{cc} \int_{-\theta_{\ell }}^{\theta_{\ell }}\frac{f^{1/2}{\tilde{k}}^{i}d\Sigma_{i}}{\omega }\; & =\int_{-\theta_{\ell }}^{\theta_{\ell }}d\theta \frac{\sqrt{M}\ell_{\mathrm{AdS}}\cosh\left(\sqrt{M}\;\theta \right)}{\sqrt{M\epsilon^{2}/\ell_{\mathrm{AdS}}^{2}+\sinh^{2}\left(\sqrt{M}\;\theta \right)}}\\ \; & =\ell_{\mathrm{AdS}}\log\left[\frac{\sqrt{\sinh^{2}\left(\sqrt{M}\;\theta_{\ell }\right)+M\epsilon^{2}/\ell_{\mathrm{AdS}}^{2}}+\sinh\left(\sqrt{M}\theta_{\ell }\right)}{\sqrt{\sinh^{2}\left(\sqrt{M}\;\theta_{\ell }\right)+M\epsilon^{2}/\ell_{\mathrm{AdS}}^{2}}-\sinh\left(\sqrt{M}\theta_{\ell }\right)}\right]\\ \; & =2\ell_{\mathrm{AdS}}\log\left[\frac{\ell_{\mathrm{AdS}}}{\left(\epsilon /2\right)\sqrt{M}}\sinh\left(\sqrt{M}\;\theta_{\ell }\right)\right]+O\left(\epsilon \right), \end{array}$$

and reproduces the “area” of the RT surface (7). Dividing by $4G_{N}$, this result correctly reproduces the EE of CFT (8). Therefore, we can regard a null geodesic congruence as one realization of the bit threads.

## 5. Conclusions

In this paper, we show that wave fronts of null rays emitted from a point on the AdS boundary are extremal surfaces in static spherical symmetric spacetimes and clarify the condition that the causal holographic information coincides with the holographic entanglement entropy. If they coincide, the RT surface can be understood as a wave front, and null rays naturally define a flow characterizing the amount of the EE of CFT. Hence, such a flow can be regarded as the bit threads.

As we assumed a point source on the AdS boundary, the shape of a region on the AdS boundary (entangling surface) becomes spherical because the boundary of the region is a wave front on the AdS boundary. However, by superposing point sources, it is possible to construct an extremal surface homologous to a region with arbitrary shapes on the AdS boundary by considering the envelope of wave fronts from each point sources. Thus, the method presented in this paper may be applicable to the plateaux problem [20,21] with non-trivial shapes of an entangling surface and to further understanding of the property of the holographic EE.

## Figures and Tables

**Figure A1:**
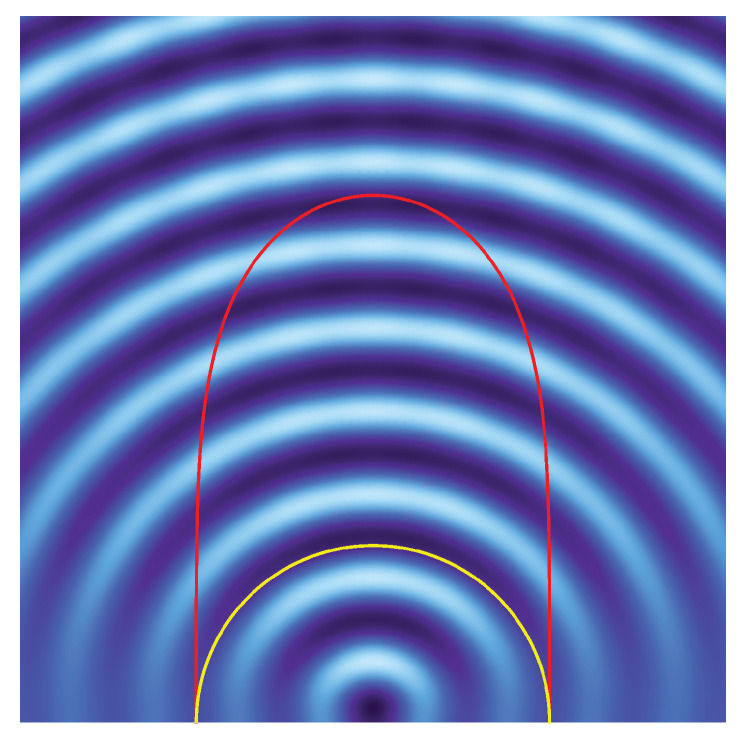
Wave pattern of the monochromatic massless scalar field in the AdS${}_{5}$ spacetime with the Poincaré patch (real part of ϕ,ω=4/3). RT surface anchoring ∂A(ℓ=10) is shown as a solid red line. The corresponding wave front (causal information surface) is shown as a solid yellow line.

## Appendix A. Massless Scalar Field in AdS Spacetimes

We consider the solution of the Klein–Gordon equation ϕ=0 in the AdS spacetime with the metric (9). Assuming the axially symmetric and stationary configuration of the scalar field $\phi \;=\;e^{-i\omega t}\tilde{\phi }\left(r,\theta \right)$, $\tilde{\phi }$ obeys the following Helmholtz type equation:

(A1) $$\frac{\partial^{2}\tilde{\phi }}{\partial r^{2}}+\left(\frac{d-1}{r}+\frac{f^{\prime }}{f}\right)\frac{\partial \tilde{\phi }}{\partial r}+\frac{\omega^{2}}{f^{2}}\tilde{\phi }+\frac{1}{fr^{2}\sin^{d-2}\theta }\frac{\partial }{\partial \theta }\left(\sin^{d-2}\theta \;\frac{\partial \tilde{\phi }}{\partial \theta }\right)=0.$$

Assuming $\tilde{\phi }=R\left(r\right)\Phi \left(\theta \right)$,

(A2) $$\begin{array}{cc} \; & R^{\prime \prime }+\left(\frac{d-1}{r}+\frac{f^{\prime }}{f}\right)R^{\prime }+\left(\frac{\omega^{2}}{f^{2}}-\frac{m(m+d-2)}{fr^{2}}\right)R=0, \end{array}$$

(A3) $$\begin{array}{cc} \; & \Phi_{\theta \theta }+\left(d-2\right)\cot\theta \;\Phi_{\theta }+m\left(m+d-2\right)\Phi =0, \end{array}$$

and $\Phi =C_{m}^{d/2-1}\left(\cos\theta \right)$ (Gegenbauer polymonial). For d=2, $\Phi =e^{im\theta },\;m\in Z$ and for d=3, $\Phi =P_{m}\left(\cos\theta \right),\;m\in Z_{+}$. We consider d=2 case. For the normalized radial function $\lim_{r\to \infty }R_{m}\left(r\right)=1$, the solution of the Klein–Gordon equation with a point source at the AdS boundary is represented as

(A4) $$\phi \left(r,\theta \right)=\sum_{m=-\infty }^{m=\infty }e^{im\theta }R_{m}\left(r\right),$$

and this wave function gives $\lim_{r\to \infty }\phi \propto \delta \left(\theta \right)$ and satisfies the boundary condition with a point wave source at the AdS boundary. For the BTZ spacetime, the solution satisfying the ingoing boundary condition at the black hole horizon is given by

(A5) $$\begin{array}{cc} \; & R_{m}=\frac{\Gamma (a)\Gamma (b)}{\Gamma (c)}\xi^{-i\frac{\ell_{\mathrm{AdS}}\omega }{2\sqrt{M}}}F\left[\frac{i}{2\sqrt{M}}\left(\ell_{\mathrm{AdS}}\;\omega -m\right),\frac{i}{2\sqrt{M}}\left(\ell_{\mathrm{AdS}}\;\omega +m\right),1+i\frac{\ell_{\mathrm{AdS}}\;\omega }{\sqrt{M}},\xi \right],\\ \; & \;a=1-\frac{i}{2\sqrt{M}}\left(\ell_{\mathrm{AdS}}\;\omega -m\right),\;b=1+\frac{i}{2\sqrt{M}}\left(\ell_{\mathrm{AdS}}\;\omega -m\right),\;c=1+i\frac{\ell_{\mathrm{AdS}}\;\omega }{\sqrt{M}}, \end{array}$$

where *F* is Gauss’s hypergeometric function and $\xi =1-M\ell_{\mathrm{AdS}}^{2}/r^{2}$. The Figure 5 and Figure 6 are obtained by taking sum in (A4) up to $m_{\max}∼200$.

In the following, we will present an example of $S_{A}\neq \chi_{A}$. We consider the 4+1 dimensional AdS spacetime with the Poincaré patch

(A6) $$ds^{2}=\frac{\ell_{\mathrm{AdS}}^{2}}{r^{2}}dr^{2}+\frac{r^{2}}{\ell_{\mathrm{AdS}}^{2}}\left(-dt^{2}+dw^{2}+dx^{2}+dy^{2}\right),$$

and consider a subregion *A* on the AdS boundary $\left\{-\ell \leq w\leq \ell ,\;\left(x,y\right)\in R^{2}\right\}$, which is a band shape region. The corresponding solution of the Klein–Gordon equation with a wave source in placed on the line w=0 is represented as

(A7) $$\phi \left(r,w,x,y\right)=\int_{-\infty }^{\infty }dp_{w}\;z^{2}N_{2}\left(\sqrt{\omega^{2}-p_{w}^{2}}\;z\right)e^{i(p_{w}w-\omega t)}$$

where $z=\ell_{\mathrm{AdS}}/r^{2}$ and $N_{2}$ is the Bessel function of the second kind. Taking the eikonal limit, the wavefronts are obtained as $w=\pm \sqrt{\ell^{2}-z^{2}},\;\left(x,y\right)\in R^{2}$. Notice that this wavefront does not coincide with the RT surface since the wavefronts are not axisymmetric and then the corresponding null congruence has the sheer. Actually, the RT surfaces of *A* are

(A8) $$w=\pm \frac{x^{d+1}\sqrt{1-{\left(x/\ell \right)}^{2d}}}{\left(d+1\right)\sqrt{\ell^{2d}-x^{2d}}}{\;}_{2}F_{1}\left(\frac{1}{2},\frac{d+1}{2d},\frac{1}{2}\left(3+\frac{1}{d}\right),\frac{x^{2d}}{\ell^{2d}}\right).$$

The RT surface coincides with the causal information surface if and only if d=2. Figure A1 depicts the wavefronts projected on t=const. slice (the causal information surfaces) and the RT surfaces corresponding to the subregion *A*.
